# Effectiveness of Counseling Based on Functional Analytic Psychotherapy with Enhanced Cognitive Therapy on the Sexual Quality of Life of Married Adolescent Women

**DOI:** 10.1055/s-0041-1733914

**Published:** 2021-08-30

**Authors:** Maryam Gholami, Atefeh Ahmadi, Mozhgan Taebi, Yunes Jahani, Katayoun Alidousti

**Affiliations:** 1 Department of Midwifery, Razi School of Nursing and Midwifery, Kerman University of Medical Sciences, Kerman, Iran; 2 Department of Midwifery, Nursing Research Center, Razi School of Nursing and Midwifery, Kerman University of Medical Sciences, Kerman, Iran; 3 Department of Anesthesiology, Faculty of Allied Medical Sciences, Kerman University of Medical Sciences, Kerman, Iran; 4 Department of Biostatistics and Epidemiology, Modeling in Health Research Center, Institute for Futures Studies in Health, Kerman University of Medical Sciences, Kerman, Iran

**Keywords:** functional analytical psychotherapy (FAP), cognitive behavioral therapy (CBT), sexual quality of life, married women, adolescents, psicoterapia analítica funcional (FAP), terapia cognitivo-comportamental (TCC), qualidade de vida sexual, mulheres casadas, adolescentes

## Abstract

**Objective** Early marriage has many deleterious effects on the health of girls, such as sexual dissatisfaction, an inevitable result of the lack of sufficient knowledge about sexual issues at the time of the marriage. The goal of the present study was to determine the effectiveness of counseling based on functional analytic psychotherapy with enhanced cognitive therapy (FECT) on the sexual quality of life of married adolescent women.

**Methods** This clinical trial was conducted between July and October 2019 on 150 married adolescent women who met the inclusion criteria. In the intervention group, FECT was conducted in sixteen 90-minute sessions twice a week. The Sexual Quality of Life-Female (SQOL-F) questionnaire was used. When the study ended, the control group was given the choice of receiving the same intervention as the intervention group.

**Results** The paired *t* -test showed a significant difference between the mean score of sexual quality of life before (52.33 ± 23.09) and after (88.08 ± 10.51) counseling in the intervention group ( *p* < 0.0001). According to the analysis of covariance, there was a significant difference between the score on sexual quality after counseling between the intervention (88.08 ± 10.51) and control (60.32 ± 23.73) groups ( *p* < 0.0001). There was also a significant difference between the mean score on the four dimensions of sexual quality of life in the intervention group ( *p* < 0.0001).

**Conclusion** The results showed that counseling based on FECT improved the sexual quality of life in all dimensions in married adolescent women.

## Resumo

**Objetivo** O casamento precoce tem muitos efeitos deletérios sobre a saúde das meninas, como a insatisfação sexual, resultado inevitável da falta de conhecimento suficiente sobre questões sexuais no momento do casamento. O objetivo do presente estudo foi determinar a eficácia do aconselhamento baseado em psicoterapia analítica funcional com terapia cognitiva aprimorada (FECT) na qualidade de vida sexual de mulheres adolescentes casadas.

**Métodos** Este ensaio clínico foi realizado entre julho e outubro de 2019 em 150 mulheres adolescentes casadas que preencheram os critérios de inclusão. No grupo de intervenção, FECT foi realizado em dezesseis sessões de 90 minutos duas vezes por semana. Foi utilizado o questionário Sexual Quality of Life-Female (SQOL-F). Quando o estudo terminou o grupo de controle teve a opção de receber a mesma intervenção do grupo de intervenção.

**Resultados** O teste t pareado mostrou diferença significativa entre o escore médio da qualidade de vida sexual antes (52,33 ± 23,09) e após (88,08 ± 10,51) o aconselhamento no grupo intervenção ( *p* < 0,0001). De acordo com a análise de covariância houve diferença significativa entre o escore de qualidade sexual após aconselhamento entre os grupos intervenção (88,08 ± 10,51) e controle (60,32 ± 23,73) ( *p* < 0,0001). Também houve diferença significativa entre a pontuação média nas quatro dimensões da qualidade de vida sexual no grupo de intervenção ( *p* <0,0001).

**Conclusão** Os resultados mostraram que o aconselhamento baseado no FECT melhorou a qualidade de vida sexual em todas as dimensões em mulheres adolescentes casadas.

## Introduction

Sexual activity is one of the most important aspects of human life. It can be influenced by personal characteristics, interpersonal relationships, the family, sociocultural conditions, the environment, the sexual-activity history of the individual and their spouse, physical and mental health, and the hormonal status of the individual. One of the most significant components of cohabitation is a healthy and satisfying sexual relationship between spouses, and having the necessary physical, mental, and social readiness is undoubtedly a necessity. 1

Sexual compatibility is an important factor in happiness and good quality of life. 2 Sexual relations can directly or indirectly affect the relationship of couples by affecting their thoughts and feelings. 3 Sexual quality of life is an interactive and dynamic state that can change over time with changes in circumstances. An optimal sex life leads to more positive sexual feelings, which, in turn, result in happiness and satisfaction with life. 4

The World Health Organization (WHO) defines the age range of 10 to 19 years as adolescence, which is one of the most important age groups in any society; therefeore, the health of adolescents is considered an important aspect of the health of society. 5

The phenomenon of early marriage is increasing in Iranian society. Early marriage has many negative consequences on health, development, and the rights of children, especially of girls, and is often accompanied by the loss of educational opportunities, social isolation, exploitation, and physical, sexual and psychological violence by their husbands. 6

Iranian cultural and religious backgrounds prevent open conversations about sexual relationships. 7 Therefore, lack of sufficient knowledge about sexual issues causes these women to feel fear and disgust regarding having sex; this sexual dissatisfaction will definitely lead to coldness in the relationship with the husband. 8

Studies 2 9 have shown that one-third of women do not enjoy sex with their partner, and almost one-fourth of them do not achieve orgasm. The prevalence of sexual dysfunction among American women ranges from 30% to 65%. 10 The prevalence of these disorders is also reported to be of 69% in Egypt, 11 and of 46.9% in Turkey. 12 The rate of sexual dysfunction is reported to be of 21.9% among newly-married women in Sari, in Iran, 13 and it has also been reported to be of 66% in newly-married women in Zanjan, also in Iran. 13

Most couples seeking divorce in Iran were young age at the time of marriage, and this is considered the most common cause for divorce. Over 50% of the causes for divorce are sexual problems, which most often occur in the first 5 years of married life. 8

Global studies show that around 82 million girls between the ages of 10 and 17 are getting married before the age of 18; 4 globally, 36% of women between the ages of 20 and 24 are married before the age of 18, with 14 million 15- to 19-year-olds giving birth each year. 8

In recent years, the highest number of marriages registered in Iran have involved men aged 20 to 24 and women aged 15 to 19; the number of recorded marriages of girls under 15 has dramatically increased in the past few years. 14

Cognitive behavioral interventions have been used to improve the sexual quality of life in men and women, 15 and studies 16 17 have reported the effectiveness of functional analytical psychotherapy on self-esteem, anxiety, depression, quality of life, and marital satisfaction.

Functional analytic psychotherapy with enhanced cognitive therapy (FECT) relies on the skills, training, forms, procedures, and methods of cognitive therapy (CT), and, compared with standard CT, experienced cognitive therapists are interested in using this method. 18

The two major enhancements FECT has brought to standard CT are the use of an expanded rationale for the causes and treatment of depression, and a greater use of the therapist-client relationship as an in-the-moment learning opportunity. In a preliminary, uncontrolled trial, 19 FECT clients appreciated the expanded rationale, the incremental improvements in depression, and the major gains in interpersonal functioning.

Despite the fact that the level of literacy in Iran is relatively high, due to gaps in the health system, the issue of counseling in sex education has been greatly ignored. 20 Considering the importance of adolescence and the high population of adolescent girls (5 million) in the country, and the fact that 17% of the country's marriages involve girls under 18 years of age, it may be possible to prevent some sexual problems in this age group. Therefore, the present study was conducted to determine the effect of the FECT approach on the sexual quality of life of married adolescent women.

## Methods

The present clinical trial (code: IRCT2019021707042736N1) was conducted to determine the effect of FECT on the sexual quality of life of married adolescent women. Sampling was conducted for 3 months, from July to the end of October 2019. The sample consisted of 150 married adolescent women who met the inclusion criteria and were referred to health care centers in Darab, a city in the south of Shiraz province, Iran. The study power was of 80%, and the significance level was 0.05. The sample size was calculated to be 80 people (40 subjects in each group), but, due to probable dropouts, we increased it by 20%.

Those who met the inclusion criteria were Iranian girls who were Darab residents, had a minimum level of literacy (reading and writing), were aged between 15 and 19 years, had been married for at least one year, had a monogamous husband (polygamy is an accepted practice in Muslim communities), had no extramarital relationships, were currently living with their husbands, had sex at least once a week, had not given birth in the previous year, reported that their spouses did not have any sexual issues, and had had an officially-registered marriage. 9 21 The exclusion criteria were having an acute illness during the study, history of mental disorders during or before the study, experiencing a stressful incident during the month preceding the study, unwillingness to continue participating in the study, use of any psychiatric or psychotropic drugs, use of any psychological services, missing at least two counseling sessions, being pregnant, history of genital surgery, and addiction to drugs or alcohol. 9 22

The eligible participants were randomly assigned to either the intervention or the control group at all eight of the health care centers in Darab. The names of the married teenagers were listed, and they were asked if they were willing to participate and if they met the inclusion criteria. A total of 150 girls met the inclusion criteria, and 75 were assigned to each group by drawing lots. Although the calculated sample size was smaller than this number, due to the large number of sessions, which increases the probability of dropouts, the study was conducted on all eligible individuals.

To facilitate the participants' commute due to the high number of counseling sessions, the intervention group was invited in groups of 10 to 12 people to the nearest health center to their residence to undergo counseling sessions. These participants completed the Sexual Quality of Life-Female (SQOL-F) questionnaire and handed it in to the researcher before the start of the first counseling session and during sessions 2, 4, 6, 8, 10, 12, 14, 15, and 16. Since each counseling session emphasized more on one aspect of the sexual quality of life, we asked the participants to fill out the questionnaire every couple of sessions to examine the differences in their answers. In the intervention group, FECT was conducted in sixteen 90-minute sessions twice a week.

The counseling sessions were based on the FECT method. To improve the efficacy of the sessions, cognitive techniques were applied to enhance the main domain of counselling (functional analytical psychotherapy, FAP). 18 19 Homework was assigned to maintain the efficacy of the sessions during the intervention; it included the repetition of the techniques and making the requested behavioral changes at home, as well as concentration on the cognitive errors to replace them with correct behaviors ( Table 1 ). The pretest and posttest were performed for both study groups simultaneously. When the study ended, the control group was given the choice of receiving the same intervention as the other group. After the posttest, an educational pamphlet containing a summary of the content of the counseling sessions was provided to the control group.

**Table 1:** Summary of the counseling sessions based on FECT

Session	Topic	Content
1	Introduction to the research variables	Introduction, explanation of the goals and rules of the counseling sessions focusing on the sexual response cycle and the benefits of sex based on the FECT approach, and homework*
2	Introduction to the method and variables	Explanation of FECT-based counseling, its benefits and limitations, types of emotions, quality of life, and sexual self-efficacy, and homework
3	Sexual disorders and self-efficacy	Analytic interpretation of and cognitive approach to women's sexual disorders and related psychological problems, the impact of morality and law on sexual quality and self-efficacy, role play, and homework
4	Sexual role and confidence, clinically-relevant behavior	Analytic interpretation of and cognitive approach to sexual roles, physical sexual attraction, sexual confidence, working on clients' clinically-relevant behavior through role play, and homework
5	Gender identity, clinically-relevant behavior	Cognitive errors about gender identity and the factors affecting it, working on the arousal of clinically-relevant behavior through role play, and homework
6	Sexual orientation, clinically-relevant behavior	Gender and sexual orientation, working on strengthening clinically-relevant behavior through role play, and homework
7	Communication skills and addiction	Working on effective marital-communication skills, behaviors that lead to sexual self-efficacy, the impact of various types of addiction on sexual relations, and homework
8	Sex life of men (husbands accompanied their wives in this session)	Cognitive focus on sexual quality of life and sexual disorders among men, and homework
9	Sexual goals	Analytic interpretation and cognitive approach to general and sexual self-efficacy and factors affecting it (such as sexual goals, self-confidence, self-esteem), and homework
10	Sexual satisfaction	Analytic interpretation and cognitive approach to sexual feelings, marital and sexual satisfaction, and their influencing factors, and homework
11	Femininity	Analytic interpretation of femininity and cognitive approach to the difference between dependence and love, how valuable women are (femininity, sexual confidence, the sense of guilt in sex life), and homework
12	Cultural factors	Analytic interpretation and cognitive approach to the cognitive triangle, sexual repression (feelings of pleasure, culture-related sexual errors), and homework
13	Improvement in sex life	Analytic interpretation of and cognitive approach to sexual competence, dealing with unexpected issues in sex life, ways to create variety in sex life, and homework
14	Role play	Interpretation of variables affecting behavior through role play, and homework
15	Review	Review session, review of practical techniques, and homework
16	Review	Review session, review of practical techniques

**Abbreviation:** FECT, functional analytic psychotherapy with enhanced cognitive therapy.

**Source:** Khajeh et al. 15 and Kanter et al. 23

**Note:** *Homework: practicing mindfulness-based changes in thoughts, emotions, and behaviors related to sexual life.

To achieve the research objectives, two questionnaires were used: a demographic questionnaire and the SQOL-F. The demographic questionnaire collected data on gender, age, duration of marriage, spouse's age, self-employment, spouse's job, number of children, level of schooling etc.

The SQOL-F was first evaluated by Symonds et al. 24 in 2005 in the United Kingdom and the United States. The internal consistency was of 0.95, and the intragroup correlation coefficient was of 0.85. The questionnaire consists of 18 questions graded on the Likert scale; each question is graded from 0 to 100 (0–20–40–60–80–100). The total score of the questionnaire is between 0 and 100. Questions 1, 5, 9, 13, and 18 are reverse-scored. The criterion for interpretation is the average score of the research population, which means that a score lower than the average of the research population indicates poor sexual quality of life, and a score higher than the average of the research population indicates the desired sexual quality of life. 25

For the statistical analysis, we used the Statistical Package for the Social Sciences (IBM SPSS Statistics for Windows, IBM Corp., Armonk, NY, US) software, version 24. As we had repetitions in the measurement, we used repeated measures analysis of variance (ANOVA) to make comparisons within the intervention group. To compare the intervention and control groups after the intervention, we used analysis of covariance (ANCOVA). The independent samples *t* -test was used to compare the study groups before the intervention due to the normality of the data, and the Chi-squared test was used to examine the similarity between both groups. The paired *t* -test was used to compare the groups before and after the intervention. To observe ethical considerations, in addition to obtaining written informed consent from the participants, the study was conducted under ethical code IR.KMU.REC.1398.091, issued by the Ethics Committee at Kerman University of Medical Sciences, and clinical trial code IRCT2019021707042736N1, issued by the Iranian Registry of Clinical Trials.

## Results

In the present study, 150 married teenage women, divided into 2 groups of 75 each, were examined, and 50 (25 women in each group) were excluded from the study. The reasons for exclusion from the intervention group were: starting school and having preparation classes for university entrance exams (ten participants); university admission and moving to another city (nine participants); pregnancy (two participants); and unwillingness to attend the meetings (one participant); in addition, three women dropped out of the study due to concomitant use of antidepressants. In the control group, some members were excluded due to failure to complete the posttest questionnaire (19 participants), or because they sent an empty questionnaire (6 participants). The pretest and posttest were competed by both study groups simultaneously. Finally, the data on 50 women in each group were analyzed.

According to the results shown in Table 2 , the mean age of the participants was 17.62 ± 1.32 years (intervention group) and 17.38 ± 1.15 years (control group) ( *p* = 0.33). The mean age of the spouse was 27.56 ± 3.43 years and 26.56 ± 3.02 years in the intervention and control groups respectively ( *p* = 0.12). There was a significant difference between the groups in terms of the number of children ( *p* = 0.04), but there was no significant difference between them regarding level of schooling, spouse's level of schooling, or spouse's job.

**Table 2:** Demographics of the study groups

Variable	Intervention group: N(%)	Control group N(%)	Chi-squared test	*p* -value
**Women education level:**			0.31	0.85
First high school	7(14)	6(12)		
Second high school	35(70)	34(68)		
Diploma and higher education	8(16)	10(20)		
**Husband's level of schooling:**			2.78	0.59
Illiterate	2(4)	0(0)		
Elementary	6(12)	4(8)		
Diploma	20(40)	20(40)		
Associate degree	10(20)	11(22)		
Bachelors	12(24)	15(30)		
**Husbandś education level:**			0.60	0.89
Unemployed	5(10)	3(6)		
Worker	5(10)	6(12)		
Freelancer	30(60)	31(62)		
Employed	10(20)	10(20)		
**Number of children**				0.04
0	27(54)	27(54)	–	
1	15(30)	15(30)	–	
2	7(14)	7(14)	–	
3	1(2)	1(2)	–	

There was no significant difference between the mean score on sexual quality of life before counseling in the intervention (52.33 ± 23.09) and control (59.57 ± 22.12) groups ( *p* = 0.11), that is, the groups were not significantly different in terms of sexual quality of life before the intervention. There was a significant difference between the mean score on sexual quality of life before (52.33 ± 23.09) and after (88.08 ± 10.51) counseling in the intervention group ( *p* < 0.0001). However, in regard to this, there was no significant difference in the control group ( *p* = 0.30). There was a significant difference between the mean score in the 4 dimensions of sexual quality of life: psychosexual aspects (84.62 ± 12.68), sexual satisfaction (93.84 ± 8.20), sexual self-humiliation (87.73 ± 12.07), and sexual repression (86.93 ± 13.46) after counseling within the intervention group ( *p* < 0.0001). However, there was no such difference in the control group ( Table 3 ). According to the ANCOVA, there was a significant difference between the score on sexual quality of life after counseling between the intervention (88.08 ± 10.51) and control (60.32 ± 23.73) groups ( *p* < 0.0001), with an average score of 31.91 (95% confidence interval [95%CI]: 27.89–35.92) in the intervention group after counseling.

**Table 3:** Comparison of the mean score on sexual quality of life and its dimensions before and after counseling in both groups

Variable	Group	Before counseling: mean ± standard deviation	After counseling: mean ± standard deviation	*p* -value
Sexual psychology	Intervention	50 ± 25.64	84.62 ± 12.68	< 0.0001
	Control	58.20 ± 23.31	59.06 ± 25.10	0.414
Sexual satisfaction	Intervention	57.68 ± 24.05	93.84 ± 8.20	< 0.0001
	Control	64.12 ± 21.94	64.79 ± 23.53	0.542
Sexual self- humiliation	Intervention	49.20 ± 24.86	87.73 ± 12.07	< 0.0001
	Control	59.46 ± 25.97	59.33 ± 27.80	0.909
Sexual repression	Intervention	52 ± 22.77	86.93 ± 13.46	< 0.0001
	Control	55.33 ± 23.26	56.80 ± 24.77	0.219
Total sexual quality of life	Intervention	52.33 ± 23.09	88.08 ± 10.51	< 0.0001
	Control	59.57 ± 22.12	60.32 ± 23.73	0.39

**Fig. 1:**
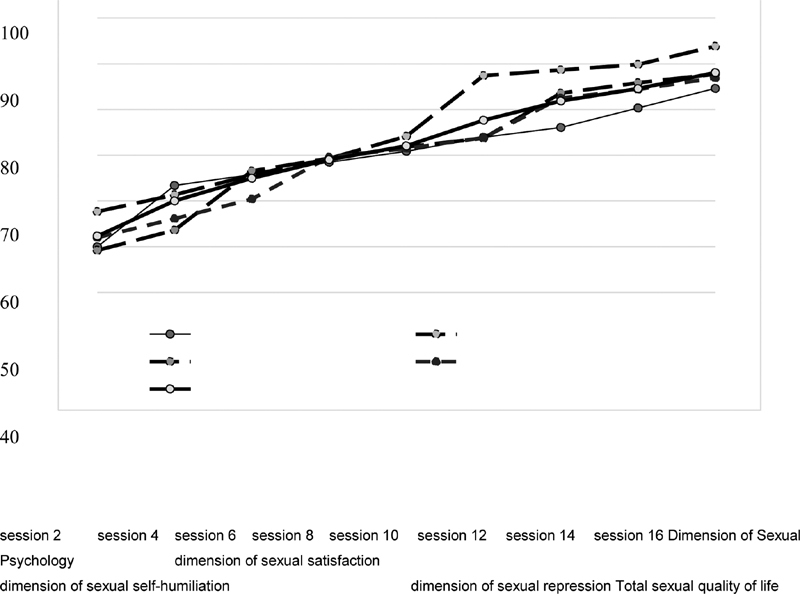
Changes in sexual quality of life and its dimensions during counseling sessions in the intervention group.

As can be seen from the repeated measures ANOVA, there was a significant difference between the average sexual quality of life and its dimensions at different times ( *p* < 0.0001) ( Table 4 ). The mean score increased over time in the intervention group. An elevation was observed in the mean score of different dimensions in different sessions. In the second and twelfth sessions, the psychological dimension improved; in the eighth session, the sexual dimension improved; sexual humiliation improved in the second and tenth sessions; and the sexual repression subscale improved in the fourth and tenth sessions ( Fig. 1 ).

**Table 4:** Trend of the average score on sexual quality of life during counseling sessions in the intervention group

Group	Intervention		
Sexual quality of life	Mean ± standard deviation	F	*p* -value *
F-test	52.33 ± 23.09	189.27	< 0.0001
Session 2	60.02 ± 22.89		
Session 4	64.97 ± 21.68		
Session 6	69.02 ± 20.05		
Session 8	72.04 ± 18.13		
Session 10	77.64 ± 15.56		
Session 12	81.88 ± 13.54		
Session 14	84.57 ± 12.10		
Session 16	88.08 ± 10.51		

Note:* Repeated measures analysis of variance (ANOVA).

## Discussion

The present study was conducted to determine the effect of FECT on the sexual quality of life of married adolescent women. The results show a significant difference regarding the mean score on sexual quality of life of both study groups ( *p* < 0.0001). A significant difference in the score on sexual quality of life after counseling was also observed for both groups through the ANCOVA ( *p* < 0.0001). This shows that FECT was effective in improving the sexual quality of life, which is consistent with the results found by Fatehi et al. 25 on the effect of psychological counseling on sexual quality of life and sexual function among breast cancer survivors in Iran. 25 Similar to the present study, the meta-analysis emphasized that individual and group psychological interventions using the cognitive approach and multidimensional therapies with long term follow-ups are suggested for the treatment of sexual dysfunction and for sexual life. 26 Another research 27 also showed that sexual rehabilitation programs have positive effects on the sexuality of patients undergoing hemodialysis. These similarities could be due to the use of psychotherapeutic interventions and counseling. Sexual rehabilitation programs, as well as FECT, increase self-esteem and the participants' ability to resume their lives as sexual beings, as their acceptance of their sexuality is addressed in rehabilitation. Furthermore, the participants' ability to resume their lives as sexual beings was affected by the their own attitudes, and those of their partners and of society. Etemadi et al. 28 conducted conducted a study with the aim of determining the effect of FAP on the rates of depression, anxiety, and marital satisfaction of women with marital issues. The results showed that FAP was effective in reducing depression and anxiety and increasing marital satisfaction. 28 The present research aimed at increasing the effectiveness of FAP by adding a cognitive element to the counselling sessions.

The results showed that there was a significant difference between the mean score on sexual quality of life before and after counseling in the intervention group ( *p* < 0.0001), that is, counseling was able to improve the sexual quality of life of the research participants. Alimohammadi et al. 29 conducted a clinical trial with a sample of 96 newly-married women with the aim of investigating the effect of counseling based on Bandura's self-efficacy theory on sexual self-efficacy and sexual quality of life. The intervention was performed in the form of six 90-minute sessions per week, and the results showed that counseling improved sexual self-efficacy, but did not affect the sexual quality of life. It seems that counseling based on Bandura's self-efficacy theory cannot cover all dimensions of sexual quality of life.

Abdelhakm et al. 30 reported that the permission, limited information, specific suggestions, and intensive therapy (PLISSIT) model sexual counseling program has a significant effect on improving the sexual quality of life of women in the postpartum period. The PLISSIT enables participants to freely discuss sexual issues in order to solve sexual problems and enhance sexual quality of life. With the FECT, these two aims are achieved by working on clinically-relevant behavior (CRB) and modifying cognitive errors and behavioral problems in sexual life to produce positive changes in those behaviors. 18

According to the results of the present study, there was a significant difference in the mean score on the four dimensions (psychosexual aspects, sexual satisfaction, sexual self-humiliation, and sexual repression) of sexual quality of life before and after counseling in the intervention group ( *p* < 0.0001). In addition, the repeated measures ANOVA showed that there was a significant difference in the average sexual quality of life and its dimensions at different times ( *p* < 0.0001). In the present study, FECT was able to have a positive effect on all aspects of sexual quality of life. Also, progress was examined in each session, and clear progress in each dimension was observed in specific sessions according to the content of that day. Ahmadian et al. 31 also obtained similar results by examining the problem-solving sexual therapy process of couples with sexual issues, demonstrating that the couples made progress in each session. Teaching problem-solving as a component of emotional intelligence was effective on enhancing sexual quality of life. 32 Both cognitive behavioral therapy (CBT) and problem-solving therapy indicated significant improvements in satisfaction over time. 33

The 2013 study by Steinke et al. 34 aimed to determine the effect of comprehensive sexual counseling based on social cognitive theory on the dimensions of sexual satisfaction, sexual self-efficacy, awareness, return to sexual activity, sexual anxiety, sexual depression, and quality of life. The study was performed on heart attack patients aged ≥ 45 years and their spouses. Educational videos, telephone counseling, and pamphlets were used in the intervention. The study was conducted on 10 patients and on the sexual partners of 3 of them. Eight weeks after the intervention, the findings showed that the participants' knowledge and information had increased. Sexual self-efficacy before and after the intervention was similar, and there was no significant difference in terms of sexual anxiety before and after the intervention. 34

This may be related to the fact that social cognitive theory (SCT) does not provide a full explanation or description of how social cognition, behavior, environment, and personality are related. 35

One study 21 showed that four 90-minute sessions of educational intervention did not cause a significant change in a woman's sense of guilt from coitus during pregnancy, of the immorality of coitus during pregnancy, and of the dislike for her pregnant appearance from the point of view of the spouse. Educational intervention, by focusing on information about psychological and physiological changes that occur during the sexual response, may not be as effective as FECT. 26

Health is one of the basic human rights, and adolescent health is a priority in most societies. There is little research on adolescent sexual problems, but evidence suggests that these problems cause concern and distress among adolescents. 22 Young people are usually less aware of sexual issues, which increases their sexual problems, and, as a result, their anxiety and worry; thus, this may create a wide range of misconceptions and false beliefs. Empowering young people by increasing their awareness regarding sexual issues can encourage them to seek professional help. 36 The high number of sessions was one of the limitations of the present research; however, the results can be used in the field of clinical and counseling services to enrich the knowledge and skills of adolescents and increase their ability to express their issues in this field.

## Conclusion

The results showed that FECT improved sexual quality of life among married adolescent women.
